# Specialized metabolic functions of keystone taxa sustain soil microbiome stability

**DOI:** 10.1186/s40168-020-00985-9

**Published:** 2021-01-31

**Authors:** Weibing Xun, Yunpeng Liu, Wei Li, Yi Ren, Wu Xiong, Zhihui Xu, Nan Zhang, Youzhi Miao, Qirong Shen, Ruifu Zhang

**Affiliations:** 1 Jiangsu Provincial Key Lab of Solid Organic Waste Utilization, Jiangsu Collaborative Innovation Center of Solid Organic Wastes, Educational Ministry Engineering Center of Resource-saving fertilizers, Nanjing Agricultural University, Nanjing, 210095 Jiangsu People’s Republic of China; 2 Key Laboratory of Microbial Resources Collection and Preservation, Ministry of Agriculture, Institute of Agricultural Resources and Regional Planning, Chinese Academy of Agricultural Sciences, Beijing, 100081 People’s Republic of China

**Keywords:** Soil incubation, Microbial diversity and stability, Co-occurrence network, Machine learning, Keystone function

## Abstract

**Background:**

The relationship between biodiversity and soil microbiome stability remains poorly understood. Here, we investigated the impacts of bacterial phylogenetic diversity on the functional traits and the stability of the soil microbiome. Communities differing in phylogenetic diversity were generated by inoculating serially diluted soil suspensions into sterilized soil, and the stability of the microbiome was assessed by detecting community variations under various pH levels. The taxonomic features and potential functional traits were detected by DNA sequencing.

**Results:**

We found that bacterial communities with higher phylogenetic diversity tended to be more stable, implying that microbiomes with higher biodiversity are more resistant to perturbation. Functional gene co-occurrence network and machine learning classification analyses identified specialized metabolic functions, especially “nitrogen metabolism” and “phosphonate and phosphinate metabolism,” as keystone functions. Further taxonomic annotation found that keystone functions are carried out by specific bacterial taxa, including *Nitrospira* and *Gemmatimonas*, among others.

**Conclusions:**

This study provides new insights into our understanding of the relationships between soil microbiome biodiversity and ecosystem stability and highlights specialized metabolic functions embedded in keystone taxa that may be essential for soil microbiome stability.

Video abstract

**Supplementary Information:**

The online version contains supplementary material available at 10.1186/s40168-020-00985-9.

## Background

The terrestrial microbiome is regarded as a ubiquitous and indispensable ecosystem component that sustains functions such as organic carbon turnover, nutrient-use efficiency, and productivity [1, 2]. Ultimately, the sustainability of both the functions and services rendered by the terrestrial ecosystem is dependent on a relatively stable microbiome [3], defined as the degree of variation or turnover rate of the microbial community [4]. These alterations in the microbiome in response to environmental changes fall into the two predominant categories of deterministic and stochastic processes [5]. Soil microbiomes encounter various natural environmental perturbations such as droughts, floods, extreme temperatures, and anthropogenic impacts like through soil contamination by antibiotics, heavy metals, and pesticides [3, 6, 7]. Although a community may respond to environmental changes through variations in the composition or dormancy of a certain microbial populations under unfavorable conditions [8], studies have identified microbiomes that exhibit compositional and functional stability over time [9, 10], while others exhibit properties of instability [11]. Therefore, it is important to understand the properties that underlie the stability of microbial assemblages as well as the possible downstream functional consequences that may have an impact on overall ecosystem function.

The stability of a microbiome has been attributed principally to species diversity since a general consensus is that biodiversity imparts positive effects on microbiome stability [12]. Species loss generally results in impaired ecosystem function [13–15]. Within the microbial community, there is evidence that keystone taxa can drive microbial community assembly [16, 17]. These keystone taxa are highly connected taxa that may exhibit unique and critical roles in organizing the structure of the soil microbiome, with downstream impacts on ecosystem processes. Thus, the removal of keystone taxa, perhaps through an environmental perturbation, may negatively impact microbiome stability and cause a dramatic shift in microbiome composition and function [18]. This potential critical role of a keystone species has been exhibited in the stability of a synthetic microbiome [19]. The microbial co-occurrence network analysis has been shown to be particularly useful in studying the complex relationships among the myriad of microbial species [20]. Network metrics such as mean degree and closeness centrality can be used to statistically identify keystone members [18]. Recently, machine learning has been implemented in order to provide deeper insight into complex microbiome interactions [21, 22], which has the potential to be used for discovering keystone taxa associated with soil microbiome stability.

Previous studies, such as those focused on the development of ecological models and the analyses of simple microbial communities with clear genetic backgrounds, have been undertaken in order to unravel intrinsic relationships between biodiversity and stability [23, 24]. However, soil microbiomes are inherently complex and harbor many diverse species that interact with one another [25], making natural systems vastly more complicated to easily study. While keystone species have been demonstrated to drive community assembly [16, 17], it remains unknown whether keystone taxa (or their respective functions) are indispensable for the stability of the larger microbiome. Experimental manipulations of soil microbiomes by the removal of putative keystone taxa and assessment of the impacts can be used to address this question. Novel approaches such as the isolation chip [26] and microbial trap [27] have been developed to study such taxa. These techniques can be used to isolate and characterize the keystone taxa, but do not allow for microbiome manipulation such as through the specific removal of keystone taxa. Fine-scale soil microcosms have the advantage of enabling the manipulation of microbial diversity by gradually removing the rare taxa from a microbiome under controlled in situ conditions [28, 29]. For instance, soil dilution has been used to show that the function of the nitrogen (N) cycle is significantly impaired by a reduction in microbiome diversity [30, 31]. This dilution-to-extinction approach can be used to explore how the function of a microbiome is linked to diversity and stability and to further study the function of keystone taxa.

In our previous study [32], we investigated the impacts of microbial α-diversity on stochastic/deterministic microbiome assembly processes. In that study, we generated a series of bacterial communities with varying levels of biodiversity by inoculating sterilized soils with a serially diluted soil suspension under various soil pH conditions. The taxonomic features and potential functional traits of the soil bacterial communities were assessed by DNA sequencing. The re-assembled bacterial communities under different pH conditions indicated that stochastic assembly processes were dominant in high-diversity communities while low-diversity triggered deterministic microbiome assembly processes were principally due to environmental filtering. This fine-scale experimental design was useful to evaluate the variations of re-assembled microbiomes from the same inoculum of different diversities under a broad range of soil pH. The results of this prior study point to the need for further analyses in order to address the relationship between soil microbiome diversity and stability. Therefore, in this study, we analyzed the prior targeted 16S rRNA gene and shotgun metagenomic sequencing data using network analysis and machine learning methods in order to identify keystone taxa and their related functional characteristics. We hypothesized that the microbial communities with high phylogenetic diversity may contain more keystone taxa and functions associated with the maintenance of soil microbiome stability.

## Methods

The experimental setup described here is based on our previous study [32], and the current work is a follow-up study to address the relationship between soil microbiome diversity and stability.

### Soil collection and microcosm incubation

The red soil was collected in August 2016 from Yingtan (116° 94′ E, 28° 21′ N), Jiangxi Province of South China. This soil is an example of Ferralic Cambisol according to the FAO/UNESCO System of Soil Classification. A fresh soil sample was obtained from the upper 20 cm of a grassland ecosystem, and soil physicochemical properties were measured [33]. Briefly, the soil pH was determined using a pH meter (PHS-3C, Shanghai, China) in a soil-to-water ratio of 1:5. The soil organic matter content was determined using the potassium dichromate volumetric method. The available nitrogen was measured by the alkaline hydrolyzable diffusion method. The available phosphorus was extracted with sodium bicarbonate and then determined using the molybdenum blue method. The available potassium was extracted with ammonium acetate and determined using flame photometry. The soil was determined to have a pH of 5.3 (± 0.3, *n* = 5) and with 1.6% (± 0.07%, *n* = 5) organic matter, 53.9 mg kg^-1^ (± 6.5 mg kg^-1^, *n* = 5) available nitrogen, 0.95 mg kg^-1^ (± 0.25 mg kg^-1^, *n* = 5) available phosphorus, and 61.5 mg kg^-1^ (± 3.5 mg kg^-1^, *n* = 5) available potassium. The soil was sieved with a 2-mm sieve and homogenized. A portion of the soil for inoculum preparation was temporarily stored (< 2 weeks) under room temperature (20 °C) and constant moisture level (30% of field capacity) in regularly aerated bags, while the rest was sterilized using γ-irradiation (> 50 kGray) (Xiyue Radiation Technology Co., Ltd., Nanjing, China) [34].

Each soil microcosm was established by placing 250 g of γ-radiation-sterilized soil into a 500-mL bottle. These microcosms were pre-incubated at 20 °C in the dark for 4 weeks, and sterile distilled water was added every 2 days to maintain a constant moisture level of 45% of field capacity. During this pre-incubation period, lime (CaO) and ferrous sulfate (FeSO_4_) were added along with sterile distilled water to generate a soil pH gradient of 4.5, 5.5, 6.5, 7.5, and 8.5. After pre-incubation, we conducted a sterility test on agar plates and a DNA extraction test using the PowerSoil DNA Isolation Kit (Mo Bio Laboratories Inc., Carlsbad, CA, USA) to confirm the sterility of these microcosm soils and the disruption of the extracellular DNA during the pre-incubation period. A 10^-1^ soil suspension was prepared by placing 20 g of fresh soil in 180 ml of sterile distilled water in a blender for 5 min. This 10^-1^ suspension was then serially diluted to create the 10^-4^, 10^-7^, and 10^-10^ suspensions. The suspension on each dilution level was fully mixed before inoculation. The 10^-1^, 10^-4^, 10^-7^, and 10^-10^ suspensions were then inoculated into the microcosms at each pH level (Additional file: Figure S1). Each treatment was replicated six times. The untreated original soil was incubated as a control. Therefore, a total of 126 microcosms were established [(5 pH levels × 4 dilution levels + 1 original soil) × 6 replicates]. All microcosms were incubated at 20 °C in the dark for 16 weeks at 45% field capacity. Soil pH was measured every 2 weeks with a pH meter (PHS-3C, Shanghai, China) at a soil-to-water ratio of 1:5.

### DNA extraction and sequencing

During incubation, we collected two replicate soil samples from each microcosm every 4 weeks. The total DNA was extracted from 0.25 g of soil using the PowerSoil DNA Isolation Kit (Mo Bio Laboratories Inc., Carlsbad, CA, USA). Three successive DNA extractions of each soil sample were pooled to minimize any DNA extraction bias. The DNA quality was assessed using a NanoDrop ND-2000 spectrophotometer (NanoDrop, ND2000, Thermo Scientific, 111 Wilmington, DE, USA), according to the 260/280-nm and 260/230-nm absorbance ratios.

16S rRNA gene abundance was investigated every 4 weeks using quantitative real-time PCR with an ABI 7500 real-time PCR system (Applied Biosystems, USA). The primers F347 (5′-GGAGGCAGCAGTRRGGAAT-3′) and R531 (5′-CTNYGTMTTACCGCGGCTGC-3′) [35] were used. The 16S rRNA gene copies in all microcosms did not increase after 8 weeks of incubation, and we found no significant changes among dilution levels and soil pH levels at the end of incubation period (Additional file: Figure S2).

With the consideration that 16S rRNA gene copies of all microcosms did not increase until the last month of the incubation period, only the DNA extractions from the 16-week-incubated samples were used for 16S rRNA gene amplicon sequencing and shotgun metagenomic sequencing. The primers for the V4 hypervariable region of the bacterial 16S rRNA gene (515F: 5′-GTGCCAGCMGCCGCGGTAA-3′ and 806R: 5′-GGACTACHVGGGTWTCTAAT-3′) [36] were used to assess the composition and diversity of the incubated bacterial communities on an Illumina MiSeq instrument (300-bp paired-end reads). The raw sequencing data was processed using the UPARSE pipeline (http://drive5.com/usearch/manual/uparse_pipeline.html) [37] with assemble paired reads (minimum 40 bp overlap between forward and reverse reads with less than 2 mismatches), quality control (quality score 30), trim length (minimum length 200 bp), dereplication, and the removal of singletons and chimeric sequences. The remaining sequences were clustered into operational taxonomic units (OTUs) at 97% similarity and the taxonomic assignment for each OTU was performed using the Silva database (Release 128) (https://www.arb-silva.de/).

The same DNA extractions for amplicon sequencing were used for shotgun metagenomic sequencing on an Illumina HiSeq2000 platform (150-bp paired-end reads). The raw sequences were quality filtered based upon a minimum Q score of 30, assembled using IDBA 1.1.1 [38], and then filtered using a minimum length of 150 bp. After quality control, all genes were predicted using MetaGeneMark v4.33 [39] and clustered at 95% similarity using CD-HIT v4.6.2 [40]. The number of reads mapping to genes for each sample was calculated using SOAPaligner 2.21 [40]. The Kyoto Encyclopedia of Genes and Genomes (KEGG, Version 58) database was used for functional gene annotation, and the bacterial phylogenetic information extracted from Nucleotide Sequence Database (Version 2020-05-15) was used for taxonomic annotation. The basic local alignment search tool (BLAST) was used for both functional gene annotation and taxonomic annotation. The detailed sequences processing could be found in the previous study [32].

### Community stability index

Soil bacterial community stability was calculated using the rarefied OTU table at 11,020 reads per sample, which was created according to the minimum sequence number of 16S rRNA gene amplicon reads per sample. Communities of the same dilution level were grouped since they were re-assembled from the same inocula, hence four sample groups were obtained for dilution levels of 10^-1^, 10^-4^, 10^-7^, and 10^-10^, respectively. Soil bacterial community stability was evaluated by average variation degree (AVD), which is calculated using the deviation degree from the mean of the normally distributed OTU relative abundance among different pH levels. Lower AVD value indicates higher microbiome stability. The variation degree for each OTU was calculated using the following equation (Eq. 1), in which ***a***_***i***_ is the variation degree for an OTU, ***x***_***i***_ is the rarefied abundance of the OTU in one sample,**‾*x***_***i***_ is the average rarefied abundance of the OTU in one sample group, and ***δ***_***i***_ is the standard deviation of the rarefied abundances of the OTU in one sample group.

1 $$ \left|{a}_i\right|=\frac{\left|{x}_i-{\overline{x}}_i\right|}{\delta_i} $$

The AVD values were calculated using the following equation (Eq. 2), in which ***k*** is the number of samples in one sample group, ***n*** is the number of OTUs in each sample group.

2 $$ \mathrm{AVD}=\frac{\sum_{i=1}^n\frac{\left|{x}_i-{\overline{x}}_i\right|}{\delta_i}}{k\times n} $$

To verify this equation, a different rarefaction depth of 8000 reads per sample and a different normalization method of DESeq variance stabilization were used to calculate the AVD values. Furthermore, the dataset from the bacterial consortium stability study of Niu et al. [19] was used to test the reliability of AVD for evaluating microbiome stability.

### Machine learning classification and 10-fold cross-validation

In order to test the importance of different microbial functional categories in structuring ecosystem functionality, we implemented machine learning classification methods. Machine learning is a serviceable technology that has demonstrated good performance in the classification of large datasets [22]. We used 10-fold cross-validation to investigate which learning algorithms should be used for functional gene classification in this study. The 10-fold cross-validation consists of dividing the training set into *z* parts, in which *z-1* parts are used as a training set and the remaining one part is used as a validation set. This method has been extensively tested and shown to provide an estimation of the true error rate [41], although the accuracy (bias and variance) has been questioned when the sample size is small [42].

In this study, the distribution of functional gene categories in all communities and their corresponding AVD values were collected as the dataset for 10-fold cross-validation. The relative abundances of functional gene categories in all communities were used as independent variables, whereas their corresponding AVD values were used as dependent variables. A total of seven machine learning classification methods (decision tree, boosting, bagging, nearest neighbor algorithm, support vector machine, random forest, and artificial neural network [43–48]) were tested (Additional file: Table S1). Average error rates were calculated to determine which method was capable of accounting for the complex interactions among gene categories. The “Random forest” method exhibited the highest accuracy combined with the lowest average error rate of 0.096, whereas the “Decision tree” method exhibited the highest average error rate of 0.186 (Additional file: Figure S3). Therefore, we evaluated the importance of individual gene categories associated with the soil microbiome AVD value using the “Random forest” method. The importance of each functional category was evaluated according to the mean decrease accuracy, which is the decreasing degree of random forest classification accuracy calculated by changing an independent variable into a random value. The change in value of a more important independent variable (functional category) will induce a greater decreasing degree of random forest classification accuracy. Therefore, the greater decline of the accuracy, the greater importance the independent variable is. The 10-fold cross-validation was performed using R statistical language and environment. The code for 10-fold cross-validation is provided in the supplementary material (Supplementary Note 1).

### Functional gene co-occurrence network analysis

The relative abundance of each functional gene category (KEGG functional gene annotated level 3) was calculated for co-occurrence network analysis. All of the calculated gene categories were from broader categories (KEGG functional gene annotated level 1) of metabolic functions, genetic information processing, organismal systems, cellular processes, and environmental information processing. Among these broad categories, the metabolic functions were further classified as broad or specialized metabolic functions. Previous studies have demonstrated that the “Sulfur metabolism” function referring to the oxidation, reduction, or disproportionation of sulfur compounds of various oxidation states to generate energy for cellular activity and growth [49] and the “nitrogen metabolism” function referring to ammonia oxidation, nitrification, or denitrification [50, 51] were restricted to specialized microorganisms. The methanogen and methane oxidizing species involved in the “Methane metabolism” function were narrowly distributed for their aerobic/anaerobic requirements or through the coupling of metabolism with nitrogen and sulfur [52, 53]. The gene clusters assigned to “Terpenoids and Polyketides metabolism” often comprise a minority of *Actinobacteria* and *Bacillus* in the soil microbial community [54]. Several investigations have demonstrated that the enzymes involved in “Xenobiotics biodegradation/metabolism” exist within a small group of microorganisms [55, 56]. Therefore, the specialized metabolic functions comprise those related to “sulfur metabolism,” “nitrogen metabolism,” “methane metabolism,” “terpenoids and polyketides metabolism,” and “xenobiotics biodegradation/metabolism” which have been shown to be limited to specialized taxa. However, other metabolic functional gene categories, including “glycolysis/gluconeogenesis,” “TCA cycle,” “pentose phosphate pathway,” “fructose and Mannose metabolism,” “galactose metabolism,” “starch and sucrose metabolism,” “pyruvate metabolism,” “glyoxylate and dicarboxylate metabolism,” “butanoate metabolism,” “propanoate metabolism,” “amino acid metabolism,” and “lipid metabolism,” are mainly related to intracellular metabolism of carbohydrates which are basic metabolic functions of living microorganisms, hence they were defined as broad metabolic functions.

A valid co-occurrence correlation was assigned between gene categories (KEGG functional gene annotated level 3) if the spearman’s correlation coefficient (*r*) was greater than 0.75 with a *P* value < 0.01 (Supplementary Note 2). The *P* values were adjusted by multiple testing corrections using the Benjamini-Hochberg’s FDR (false discovery rate) method to reduce the chance of false-positive results [57]. Topological characteristics were calculated to describe the complexity of gene co-occurrence networks, including average degree (avgK, which is a key topological property to describe how well a node is connected to the others, higher avgK value means a more complex network), average clustering coefficient (avgCC, which is used to measure the extent of module structure present in a network), average path distance (APD, which is the average value of the distances between every two nodes in a network, higher APD value means a reduced coupling among nodes in a network), modularity (M, which is calculated to measure how well a network is able to be separated into modules), and graph density (GD, which is closely related to the average degree). Each network was statistically compared to an identically sized Erdös-Réyni random model [58]. The topological role of each gene was determined according to the *Zi* degree (how well a node is connected to other nodes in the same module) and *Pi* degree (how well a node is connected to the nodes in other modules) [59]. According to the suggested *Zi* and *Pi* degree thresholds [60], all genes were categorized into four subcategories: peripherals (*Zi* ≤ 2.5 and 0 ≤ *Pi* ≤ 0.62), connectors (*Zi* ≤ 2.5 and *Pi*> 0.62), module hubs (*Zi*> 2.5 and *Pi* ≤ 0.62), and network hubs (*Zi*> 2.5 and *Pi*> 0.62). Overall, the correlations were calculated using the psych package (version 1.8.12) [61] in R software (version 4.0.2). The networks were visualized, and the topological characteristics were calculated using Gephi software (version 0.9.2) [62].

### Statistical analysis

The phylogenetic diversity index (alpha-diversity) calculation was performed based on the rarefied OTU table using the vegan R package [63]. Phylogenetic community dissimilarity was calculated by FastUnifrac using the normalized percent frequency based on the rarefied OTU table [64].

We calculated the mean nearest taxon distance metric using the picante R package [65] and implemented a previously developed null modeling approach to calculate the β-nearest taxon index (βNTI) to infer the community assembly processes [5]. A βNTI > 2 is considered the result of the variable selection of deterministic processes with significantly more phylogenetic turnover than expected. A βNTI ≤ 2 is considered the result of the homogeneous selection of deterministic processes with significantly less phylogenetic turnover than expected. Values of |βNTI| less than 2 indicate that the differences in phylogenetic composition are the result of stochastic processes [5]. The R code for calculating βNTI metrics is provided in the supplementary material (Supplementary Note 3).

For the metagenomic DNA sequencing data, the percent frequency of one functional category was defined as the number of sequences affiliated with that category divided by the total number of sequences per sample. This normalized metagenomic data was utilized for the heatmaps in order to visualize the functional genes that were enriched or depleted within each functional category. Data were normalized by subtracting the mean and dividing by the standard deviation. Duncan’s multiple comparisons tests were used to calculate significant differences among samples while Tukey’s HSD tests were used to calculate the significance between two samples. All correlations were calculated using Spearman correlations. All statistical analyses were performed using R software (version 4.0.2; https://www.r-project.org/).

## Results

### Dilution reduces the alpha-diversity and stability of soil bacterial community

Taxonomic abundance-based Weighted UniFrac community dissimilarity between bacterial communities of different dilution levels and the un-diluted initial soil showed significantly larger variances with increased dilution (Additional file: Figure S4). However, the dissimilarity between the 10^-1^ diluted samples and the undiluted initial soil samples exhibited no significant changes in dissimilarity within the undiluted initial soil samples. Considering that manipulation of the soil pH was not performed to the undiluted initial soil sample along with the finding of no significant differences with the re-assembled 10^-1^ diluted bacterial communities, we only analyzed the diluted and re-assembled communities in this study.

**Fig. 1:**
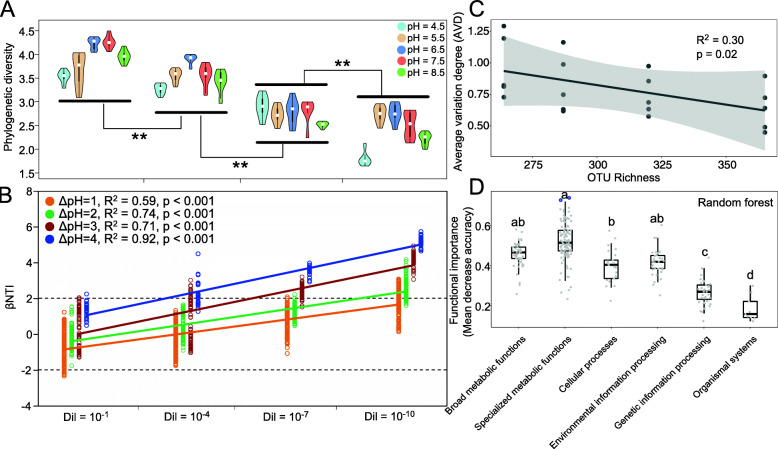
Diversity and variations of the re-assembled bacterial communities. **a** The variances of bacterial phylogenetic diversity indices across soil pH and dilution gradients. **b** The relationship between βNTI and dilution level. ΔpH is the differences in soil pH. **c** The relationship between bacterial OTU richness and average variation degree (AVD) of re-assembled bacterial communities. Asterisks indicate significance: **P value < 0.01 based on two-sided tests. Lg(Dil): Lg transformed dilution level. **d** The importance of functional categories based on random forest method (mtry = 9, ntree = 660) with a OOB (out-of-bag) estimate of error rate of 8.75%. Different letters indicate significant differences (*P* value < 0.05) between functional categories according to Duncan’s multiple comparison. The top two points of specialized metabolic functions labeled by blue outer rings indicate the functions of “nitrogen metabolism” (mean decrease accuracy values = 0.736) and “phosphonate and phosphinate metabolism” (mean decrease accuracy values = 0.749)

Dilution imposed significant effects on soil bacterial alpha-diversity (Fig. 1a). Phylogenetic diversity was highest in the least diluted soils and was significantly reduced (*P* value < 0.01 based on Tukey’s HSD test) with increased dilution. We identified significant positive correlations between βNTI values and differences in pH (Spearman’s correlation coefficient *R*^2^ ≥ 0.35, *P* value < 0.001, two-sided tests) at each dilution level (Additional file: Figure S5A and Supplementary dataset 1). When all dilutions were combined, the relationship between βNTI and differences in soil pH remained significant (Spearman’s correlation coefficient *R*^2^ = 0.23, *P* value < 0.001, two-sided tests, Additional file: Figure S5B). Under the same differences in soil pH, dilution levels were positively correlated with βNTI (Spearman’s correlation coefficient *R*^2^ ≥ 0.59, *P* value < 0.001, two-sided tests, Fig. 1b).

The AVD values increased with increasing dilution level regardless of sample depth (11,020 or 8000 reads per sample, Additional file: Supplementary datasets 2 and 3) or when a different normalization method of DESeq variance stabilization was used (Additional file: Figure S6 to S8). In order to verify the reliability and application of the AVD index as an indicator of microbiome stability, we calculated the AVD values of different bacterial consortia from the study of Niu et al. [19], in which a seven-strain synthetic community [*Enterobacter cloacae* (Ecl), *Stenotrophomonas maltophilia* (Sma), *Ochrobactrum pituitosum* (Opi), *Herbaspirillum frisingense* (Hfr), *Pseudomonas putida* (Ppu), *Curtobacterium pusillum* (Cpu), and *Chryseobacterium indologenes* (Cin)] was constructed and the community stability was assessed by the detection of community composition after the removal of one species within the original community (Additional file: Supplementary dataEx1). Niu et al. [19] found that *E. cloacae* (Ecl) was critical in maintaining the stability of this synthetic bacterial community. We found that the AVD value was the highest when *E. cloacae* was removed from the community (Additional file: Figure S9). This suggested that AVD was suitable to indicate microbiome stability.

When these datasets were divided according to soil pH within each dilution level, low-AVD values were identified in soils that were close to neutral pH (Additional file: Figure S6, S7 and S10). To evaluate the contribution of species richness to the stability of the bacterial community, we examined the relationship between AVD values (in 11,020 reads per sample of rarefaction depth) and bacterial species richness. The AVD values were found to be negatively correlated with species richness (Spearman’s correlation coefficient *R*^2^ = 0.47, *P* value = 0.0009, Fig. 1c).

To elucidate the relationship between functional genes and microbiome stability, evaluation of the importance of gene categories associated with AVD values was conducted by the “random forest” method (Additional file: Supplementary dataEx2). The importance of a proportion of specialized metabolic functions were higher (mean decrease accuracy values > 0.6) than those of other functional categories (Fig. 1d). Among these, “nitrogen metabolism” (mean decrease accuracy values = 0.736) and “phosphonate and phosphinate metabolism” (mean decrease accuracy values = 0.749) were deemed the most important functions. Therefore, the change in relative abundance of the overall specialized metabolic functions may induce a greater decline of mean decrease accuracy value. This suggests that lower AVD values are associated with a higher abundance of specialized metabolic functions, followed by functional categories of broad metabolic functions, environmental information processing, and finally, cellular processes.

### Dilution simplifies the functional gene co-occurrence network

**Fig. 2:**
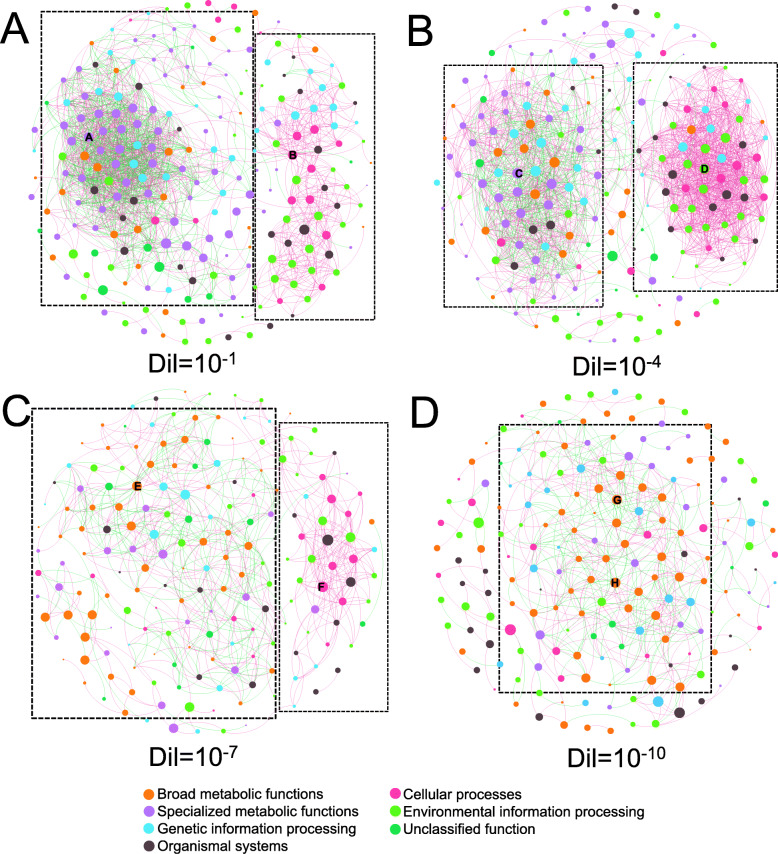
Functional gene co-occurrence networks at every dilution level. Valid co-occurrence of functional genes with strong (Spearman’s correlation coefficient *r* > 0.75) and significant (*P* value < 0.01) correlations at 10^-1^ (**a**), 10^-4^ (**b**), 10^-7^ (**c**), and 10^-10^ (**d**) dilution levels. Color points (nodes) are gene categories (KEGG functional gene annotated level 3). The size of each point is the node degree (proportional to the number of connections). A blue connection (edge) between two nodes indicates a negative correlation, while a red edge indicates a positive correlation. The capitals inside the nodes are module hubs in the individual network. Dotted line frames encompass the gene aggregates. The left frame shows the gene aggregate of “metabolic processes,” and the right frame shows the gene aggregate of “environmental signal responses” for each network (only one gene aggregate was observed in the 10^-10^ diluted network). The nodes labeled by letters (A to H) are keystone genes, and the details for these keystone genes are listed in Table S2

We sought to determine functional gene co-occurrence patterns using network analyses based on strong and significant correlations (Spearman’s correlation coefficient *r* > 0.75, *P* value < 0.01, two-sided tests) at each dilution level. Modularity indices decreased from 0.680 to 0.579 with dilution (Table 1). Despite the impact of dilution on the overall functional gene co-occurrence patterns (Fig. 2), the individual networks could be divided into two main gene aggregates. This was especially true in the less diluted networks. We defined one gene aggregate as “metabolic processes” which was primarily comprised of genes encoding broad and specialized metabolic functions. The other gene aggregate was defined as “environmental signal responses” that contained genes related to cellular processes and environmental information processing. The remaining functional categories, genetic information processing, and organismal systems were not specifically present in either gene aggregate. It should be noted that there was only one gene aggregate in the 10^-10^ diluted network.

**Table 1:** The topological properties of the functional gene co-occurrence networks on four dilution levels and their respective identically sized random networks

Network indexes	Dilution			
	Dil = 10^-1^	Dil = 10^-4^	Dil = 10^-7^	Dil = 10^-10^
*Empirical Networks*				
Total nodes	231	205	184	183
Total links	1844	1993	736	603
Metabolic process (positive-negative)	718–712	532–394	269–276	293–277
Environmental signal responses (positive-negative)	368–4	893–8	188–1	–
Average degree (avgK)	17.67	18.39	8.01	7.59
Average clustering coefficient (avgCC)	0.647	0.662	0.586	0.538
Average path distance (APD)	4.22	3.39	4.64	4.71
Modularity (M)	0.679	0.680	0.625	0.579
Graph density (GD)	0.168	0.171	0.088	0.072
*Random Networks*				
Average clustering coefficient (avgCC)	0.202 ± 0.008	0.198 ± 0.007	0.113 ± 0.007	0.153 ± 0.014
Average path distance (APD)	2.499 ± 0.025	2.365 ± 0.021	2.602 ± 0.021	2.729 ± 0.023
Modularity (M)	0.265 ± 0.004	0.258 ± 0.005	0.232 ± 0.006	0.223 ± 0.005

For all networks, the average clustering coefficient, average path distance, and modularity indices in the empirical networks were larger than those of their respective identically sized random networks (Table 1). Dilution exhibited a strong impact with a reduction in the total nodes (functional genes) and links (positive or negative correlations). The average degree, average clustering coefficient, modularity, and graph density indices were lower in the more diluted networks, indicating that dilution reduced the complexity of the re-assembled soil microbiome. These indices also exhibited a slight increase in the 10^-4^ diluted network, compared to the 10^-1^ diluted network. In addition, the average path distance followed an opposite trend. In summary, dilution reduced the complexity of gene co-occurrence networks.

For the gene aggregate “metabolic processes,” although the number of positive and negative correlations both decreased with increasing dilution, we observed that the number of positive correlations was nearly identical to that of negative correlations at every dilution level (Table 1). Notably, specialized metabolic functional genes were dominant in the less diluted networks, whereas broad metabolic functional genes became dominant in the more diluted networks (Fig. 2). The majority of negative correlations were identified to be between the broad metabolic functional and the specialized metabolic functional genes, while most of the positive correlations were detected between genes within either the broad or specialized metabolic functions. Furthermore, the gene aggregate of “environmental signal responses,” in which most of the co-occurred genes were positively correlated (Table 1), gradually became less complex with increasing dilution. Lastly, the gene aggregations of “metabolic processes” and “environmental signal responses” were not observed in the most diluted network (Fig. 2d).

### Keystone microorganisms devoted to specific functions facilitate the stability of soil microbiome

According to the connection degree of individual node within (*Zi* degree) and among (*Pi* degree) modules, we evaluated the topological roles of all functional categories and defined the keystone functions within each network (Additional file: Table S2). All of these keystone functional categories were also detected as module hubs. In the less diluted networks (10^-1^ and 10^-4^), the nodes of “nitrogen metabolism” and “phosphonate and phosphinate metabolism” in specialized metabolic functions were keystone functions within the “metabolic processes” gene aggregate. Keystone nodes corresponding to “adherens junction” in cellular processes and “ion channels” in environmental information processing were observed in the “environmental signal responses” gene aggregate. In contrast, in more diluted networks (10^-7^ and 10^-10^), the keystone nodes of “citrate cycle,” “glycolysis/gluconeogenesis,” and “starch and sucrose metabolism” in broad metabolic functions and “bacterial chemotaxis” in cellular processes were identified.

**Fig. 3:**
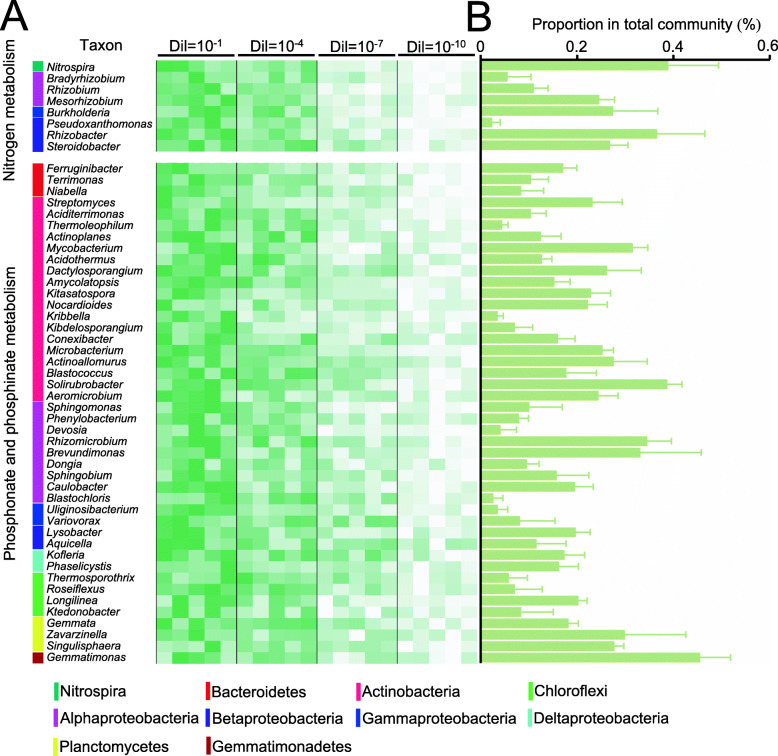
Taxonomic annotation of the two keystone functions. **a** Relative abundances of the 8 genera for “nitrogen metabolism” and 44 genera for “phosphonate and phosphinate metabolism” across soil pH and dilution levels, based on shotgun metagenomic data. The relative abundances of genera at each row were normalized by removing the mean and dividing by the standard deviation. The color from green to white represents a relative abundance of each genus from high to low. Five columns at each dilution level within the heatmap indicate the soil pH range of 4.5 (left) to 8.5 (right). The genera are colored by phylum on the left side. **b** The relative abundances of the corresponding genera based on shotgun metagenomic data. Error bars represent standard deviations (SD, *n* = 60)

Since the specialized metabolic functions of “nitrogen metabolism” and “phosphonate and phosphinate metabolism” were defined as the most important functions by the random forest classification method and by the identification of keystone nodes in the functional gene co-occurrence network analysis that are both associated with microbiome stability, we then performed taxonomical annotation to identify the related taxa. A total of 8 genera for “nitrogen metabolism” and 44 genera for “phosphonate and phosphinate metabolism” were identified (Fig. 3 and Additional file: Supplementary dataset 4). All these genera were more abundant in less diluted samples with a higher impact imparted by dilution than pH (Fig. 3a). *Nitrospira* was the most frequently detected genus contributing to soil nitrogen metabolism, followed by *Rhizobacter*, *Mesorhizobium*, *Steroidobacter*, and *Burkholderia* affiliated with the *Proteobacteria* (Fig. 3b). In addition, *Gemmatimonas*, affiliated with the *Gemmatimonadetes*, was the most abundant genus involved in phosphonate and phosphinate metabolism, followed by *Solirubrobacter* within *Actinobacteria*, and *Brevundimonas* and *Rhizomicrobium* within *Proteobacteria*. In comparison, although some of the above identified genera were not detected by amplicon sequencing (Additional file: Figure S11), we found the abundant genera in functional gene annotation, such as the genera *Nitrospira*, *Rhizobacter*, and *Burkholderia* associated with soil nitrogen metabolism and the genera *Gemmatimonas* and *Brevundimonas* associated with phosphonate and phosphinate metabolism, were also detected in high frequencies. This supports a general consistency of these two datasets for the identification of keystone microbes.

## Discussion

The relationship of biodiversity and ecosystem stability in microbial ecosystems has been debated for many years within the field of theoretical ecology by using interaction models [25] or artificially cultivated simplified systems consisting of only a few species [66]. Due to the high complexity, high species diversity, and intractability of the soil microbiome, appropriate experiments that address this ecological theory in soil microbial ecosystems remain less explored. In general, the soil microbiome is characterized by functional redundancy, suggesting a moderate reduction in the abundance of any taxa will likely result in a marginal impact on the overall function of the soil microbiome since other bacteria can also perform an identical function [67]. However, an intensive diversity loss will induce impaired ecosystem functioning [30, 31].

Dilution reduced the bacterial phylogenetic diversity in the inocula. Consequently, this reduced diversity should significantly impact the soil microbial assembly processes [32]. The identified relationships between βNTI and dilution level and difference in soil pH indicated that there is a transition in the process of soil microbiome assembly from significantly less than expected phylogenetic turnover to significantly more than expected phylogenetic turnover as the dilution level or difference in soil pH increases [5]. Therefore, this result revealed that variations in the soil microbiome among different pH levels were greater at higher dilution levels.

Microbiome stability can be described by the resilience (the community recovery process to an alternative stable state) or the resistance (the community remains unchanged in response to a disturbance) of a community [68]. Thus, microbiome stability may be evaluated by comparing variations in microbiome composition/function facing when challenged with different environmental conditions, e.g., temperature, soil pH [69]. If a community is sufficiently stable, there will not be large compositional or functional variations under sustained environmental perturbation. Here, we developed a new strategy to study the stability of the soil microbiome by calculating AVD values based on our experimental design. This strategy is not limited by the number of samples and has advantages in evaluating the community stability over other methods, such as calculating fold changes [70] and community distances [19], which can only be used to compare two samples at a time. As expected, in this study, lower AVD values exhibited lower variation in the less diluted samples that harbored higher bacterial phylogenetic diversity. Moreover, higher phylogenetic diversity often occurred near neutral pH and corresponded to lower AVD values. As such, smaller variations were associated with higher microbial phylogenetic diversity in the microbiome, indicating the positive relationship between the stability and biodiversity of soil microbiome.

Network analysis can provide comprehensive insights into the microbiome and disentangle co-occurrence patterns on both the phylogenetic and functional levels [71]. We observed that the functional gene co-occurrence networks were scale-free and matched the behavior of a small-world with intrinsic modular architecture due to a larger average clustering coefficient, average path distance, and modularity indices as compared to identically sized random networks [59]. Dilution exerted a remarkable effect on soil functional gene co-occurrence networks in terms of average degree, average path distance, and modularity. Lower average degree, graph density, and average clustering coefficient values coupled with increased average path distance in the more diluted networks illustrated the reduced coupling among the dominant functional nodes. The 10^-4^ diluted network was unique in that the number of total nodes was lower but there were slightly higher values of average degree, graph density, and average clustering coefficient, compared to the 10^-1^ diluted network. This suggests that a moderate diversity loss may increase the metabolic efficiency of the soil microbiome despite a decline in microbial diversity [25, 70]. As higher modularity has been suggested as being essential for increasing the stability of a network and promoting the stability of a microbiome [72], the diluted soil microbiome that exhibited low modularity indicated an unstable community. This is coupled with higher AVD values that were associated with lower bacterial phylogenetic diversity.

Our previous study [32] demonstrated that dilution reduced the functional diversity of both the specialized and broad functions synchronously, with a decrease in the relative abundance of specialized metabolic functional genes and an increase in that of broad metabolic functional genes. Here, the majority of negative correlations were observed between the specialized and broad metabolic functional genes, which are likely due to the opposite trends in relative abundances of these two types of genes with serial dilution. A recent study [73] suggested that soil microbiome diversity and the associated microbial network complexity are positively related to ecosystem multifunctionality because the functional redundancy decreased when microbial diversity was low, especially for the functions that were only supported by few taxa. Broad metabolic functions are usually associated with intracellular metabolism that can be accomplished within a single cell. However, specialized metabolic functions may involve multiple steps by a series of functionally or taxonomically specific microbial species that require environmental informational sensing and cell membrane transport [74, 75]. Consequently, dilution decreased the microbial diversity and reduced the occurrences of specialized metabolic functional genes which appear to have negative impacts on ecosystem multifunctionality and microbiome stability.

We analyzed the metagenomic dataset with diverse classification methods and found that the random forest classification method, which is widely used in soil microbial ecology [76, 77], was the most accurate in identifying the importance of all functional categories. In addition, the highly connected members are keystone members in co-occurrence network [78]. Interestingly, both the random forest classification and functional gene co-occurrence network analyses demonstrated that the specialized metabolic functions of “nitrogen metabolism” and “phosphonate and phosphinate metabolism” were the keystone functions associated with the AVD values and likely contributed to microbiome stability.

Taxonomic annotation of the keystone functions of “nitrogen metabolism” and “phosphonate and phosphinate metabolism” demonstrated that a group of bacterial genera could possibly be keystone species associated with microbiome stability. The majority of these bacteria were identified within the phyla *Actinobacteria* and *Proteobacteria*. Previous studies based on phylogenetic network analysis of 16S rRNA gene amplicon sequencing data have also found that the majority of bacterial keystone taxa were members of the *Proteobacteria* and *Actinobacteria* [79, 80]. Therefore, the keystone taxa identified by the functional gene co-occurrence networks and machine learning analyses based on shotgun metagenomic sequencing data were consistent with those identified from amplicon sequencing data.

Not surprisingly, the function of “nitrogen metabolism” was detected to be a keystone function containing the potential keystone genera of *Nitrospira*, *Rhizobacter*, *Mesorhizobium*, *Steroidobacter*, and *Burkholderia*. *Nitrospira* are ammonia- and nitrite-oxidizing bacteria [81, 82], whereas *Rhizobacter*, *Mesorhizobium*, and *Burkholderia* comprise nitrogen-fixers [83, 84]. Additionally, *Steroidobacter* is known to be related with denitrification processes [85]. Interestingly, numerous studies have revealed that nitrogen cycling taxa are consistently identified as keystone taxa across diverse ecosystems [80, 86, 87]. In contrast, the function of “phosphonate and phosphinate metabolism” is less well documented. However, the most abundant *Gemmatimonas* genus is one of the dominant groups identified in agricultural ecosystems [16]. Other genera such as *Solirubrobacter*, *Brevundimonas*, and *Rhizomicrobium* have also been identified as keystone taxa [86, 88, 89], many of which contain broad metabolic functions relating to soil organic carbon turnover [16]. However, there is no evidence concerning the specialized metabolic functions of these keystone taxa.

It has been proposed that the importance of keystone taxa may be related to the broadness of a process, referring to how many steps or diverse microbial groups are involved [18]. In this study, dilution reduced the overall bacterial phylogenetic diversity and the relative abundance of the keystone functions and taxa. This was coupled with a gradual reduction in co-occurrence network complexity and decreased microbiome stability. A previous study has suggested that a decline in soil microbial diversity does not influence the stability of soil key microbial functional groups [9]. However, in that study, the functional groups were not so narrowly distribute, and the dilution level was not so intensive such that the redundancy of the soil functions was likely not depleted. Compared to the broad processes, such as carbohydrate metabolism within the dominant taxa, the specialized metabolic functions consisting of narrow processes (for instance, nitrogen fixation, or ammonia oxidation that only consisting of a single step) devoted to a small group of specific microorganisms will be more pronounced when the keystone taxa are interfered with [30]. Therefore, the narrowly distributed keystone components of a complex community are associated with different types of ecosystem functions [90]. We speculate that the abundant bacterial genera that include *Nitrospira*, *Rhizobacter*, and *Burkholderia* referring to the keystone functions of “nitrogen metabolism” and the abundant genera that include *Gemmatimonas* and *Brevundimonas* referring to the keystone functions of “phosphonate and phosphinate metabolism” are pivotal taxa that may contribute to soil microbiome stability.

## Conclusions

Our results indicated that dilution significantly reduced phylogenetic diversity, simplified the modularity of functional gene co-occurrence networks, and decreased the soil microbiome stability. We highlighted that the specialized metabolic functions of “nitrogen metabolism” and “phosphonate and phosphinate metabolism” devoted to the abundant specific bacterial keystone taxa like *Nitrospira* and *Gemmatimonas* were associated with soil microbiome stability. Such keystone functions and taxa may provide further insight into the prediction of microbial community shifts and increase the possibility of manipulating the function of the microbiome. The possible contribution of keystone taxa to microbiome stability could then be harnessed to improve ecosystem services.

## Supplementary Information

**Additional file 1: Figure S1**. (A) Schematic diagram of soil microcosm establishment. (B) Soil incubation experimental design. **Figure S2**. The Lg transformed 16S rRNA gene copies in all microcosms at the end of incubation period (sixteen weeks) by quantitative real-time PCR. Error bars represent standard deviations (SD, n = 12). Dil: Dilution level. No statistically significant differences were found among different dilutions and pH levels. **Figure S3**. The average error rate of 10-fold cross validation based on seven machine learning classification methods. Error bars represent standard deviations (SD, n = 12). Different letters above bars indicate significant differences (*P-*value < 0.05) between machine learning methods according to Duncan’s multiple comparison. **Figure S4**. Weighted Unifrac distances between bacterial communities from different dilution levels and the untreated initial soil (CK). Lg(Dil) indicates the Lg transformed dilution level. Lg(Dil) = 0 represents the untreated soil. Asterisks indicate significance: **, *P-*value < 0.01 based on Tukey’s HSD test. n.s.: Not significant. Boxplot: median, 25%/75% percentiles, and the highest, lowest and extremely values are shown. **Figure S5**. The relationship between βNTI and differences in soil pH on each dilution level (A) and across all samples (B). **Figure S6**. The average variation degree (AVD) of all OTUs on each dilution level. Horizontal axis represents different OTUs and vertical axis represents the absolute AVD value of each OTU. **Figure S7**. The average variation degree (AVD) values under different rarefaction depths of 11,020 reads and 8,000 reads per sample. **Figure S8**. The average variation degree (AVD) values under the normalization method of DESeq variance stabilization. **Figure S9**. The average variation degree (AVD) values of a community stability test (ref [19] of the main text) by detecting the dynamics of community composition with one of the species removed from the original seven-strain synthetic community. The seven strains are *E. cloacae* (Ecl), *S. maltophilia* (Sma), *O. pituitosum* (Opi), *H. frisingense* (Hfr), *P. putida* (Ppu), *C. pusillum* (Cpu), and *C. indologenes* (Cin). C7, all seven strains; -Cpu, remove *C. pusillum* from seven strains; -Cin, remove *C. indologenes* from seven strains; -Opi, remove *O. pituitosum* from seven strains; -Hfr, remove *H. frisingense* from seven strains; -Ecl, remove *E. cloacae* from seven strains; -Ppu, remove *P. putida* from seven strains; -Sma, remove *S. maltophilia* from seven strains. **Figure S10**. The average variation degree (AVD) of all OTUs on each pH level for 10^-1^ (A), 10^-4^ (B), 10^-7^ (C) and 10^-10^ (D) diluted samples. Horizontal axis represents different OTUs and vertical axis represents the absolute AVD value of each OTU. **Figure S11**. The relative abundance of the same bacterial genera in Fig. 3 detected by 16S rRNA gene amplicon sequencing. The genera with red text were not detected in amplicon sequencing. Error bars represent standard deviations (SD, n = 240). **Table S1**. The packages and functions of machine learning classification methods. **Table S2**. The nodes identified as module hubs in the functional gene co-occurrence networks on four dilution levels. **Supplementary Note 1**. R codes for 10-fold cross validation using seven machine learning classification methods. **Supplementary Note 2**. R codes for calculating spearman’s correlation between gene categories. **Supplementary Note 3**. R codes for calculating βNTI metrics.

**Additional file 2: Supplementary dataset 1**. Results of null modeling analysis. **Supplementary dataset 2**. The relative abundance and variation degree of each OTU in every treatment. **Supplementary dataset 3**. The average variation degree (AVD) valves on different rarefaction depths of sequencing reads. **Supplementary dataset 4**. The relative abundance of every functional group by taxonomic annotation of the shotgun metagenomic sequencing data.

**Additional file 3: Supplementary dataEx1**. The community composition and the corresponding AVD values of different bacterial consortia from the study of Niu *et al.* [19].

**Additional file 4: Supplementary dataEx2**. An example dataset for calculating the functional importance using random forest classification method.

**Additional file 5:.** The example data files and R codes for calculating βNTI metrics.

## Data Availability

The “vegan,” “picante,” and “psych” are packages for the R statistical language and environment. The codes for vegan (http://vegan.r-forge.r-project.org), picante (http://picante.r-forge.r-project.org), and psych (https://CRAN.R-project.org/package=psych) are freely available on the web. The R codes are provided in Additional file. The DNA sequences from all incubation samples are deposited in the NCBI Sequence Read Archive (SRA) database with accession numbers of SRR8857587, SRR8857588, SRR8857589, SRR8857590, SRR8857591, and SRR8840928.
