# Supplementary material for: Specialized metabolic functions of keystone taxa sustain soil microbiome stability
Source: Microbiome. 2021 Jan 31;9:35. doi: 10.1186/s40168-020-00985-9 (PMC7849160; doi:10.1186/s40168-020-00985-9)
Supplement: Supplementary file 2 — Additional file 1: Figure S1. (A) Schematic diagram of soil microcosm establishment. (B) Soil incubation experimental design. Figure S2. The Lg transformed 16S rRNA gene copies in all microcosms at the end of incubation period (sixteen weeks) by quantitative real-time PCR. Error bars represent standard deviations (SD, n = 12). Dil: Dilution level. No statistically significant differences were found among different dilutions and pH levels. Figure S3. The average error rate of 10-fold cross validation based on seven machine learning classification methods. Error bars represent standard deviations (SD, n = 12). Different letters above bars indicate significant differences (P-value < 0.05) between machine learning methods according to Duncan’s multiple comparison. Figure S4. Weighted Unifrac distances between bacterial communities from different dilution levels and the untreated initial soil (CK). Lg(Dil) indicates the Lg transformed dilution level. Lg(Dil) = 0 represents the untreated soil. Asterisks indicate significance: **, P-value < 0.01 based on Tukey’s HSD test. n.s.: Not significant. Boxplot: median, 25%/75% percentiles, and the highest, lowest and extremely values are shown. Figure S5. The relationship between βNTI and differences in soil pH on each dilution level (A) and across all samples (B). Figure S6. The average variation degree (AVD) of all OTUs on each dilution level. Horizontal axis represents different OTUs and vertical axis represents the absolute AVD value of each OTU. Figure S7. The average variation degree (AVD) values under different rarefaction depths of 11,020 reads and 8,000 reads per sample. Figure S8. The average variation degree (AVD) values under the normalization method of DESeq variance stabilization. Figure S9. The average variation degree (AVD) values of a community stability test (ref [19] of the main text) by detecting the dynamics of community composition with one of the species removed from the original seven-strain synthetic commu [file 40168_2020_985_MOESM1_ESM.pdf]

Supplementary Information

Specialized metabolic functions of keystone taxa sustain soil microbiome stability

Authors:

Weibing Xun[1§], Yunpeng Liu[2§], Wei Li[1], Yi Ren[1], Wu Xiong[1], Zhihui Xu[1], Nan Zhang[1], Youzhi Miao[1], Qirong Shen[1], Ruifu Zhang[1, 2*]

Affiliations:

[1]Jiangsu Provincial Key Lab of Solid Organic Waste Utilization, Jiangsu Collaborative Innovation Center of Solid Organic Wastes, Educational Ministry Engineering Center of Resource-saving fertilizers, Nanjing Agricultural University, Nanjing 210095, Jiangsu, P.R. China

[2]Key Laboratory of Microbial Resources Collection and Preservation, Ministry of Agriculture, Institute of Agricultural Resources and Regional Planning, Chinese Academy of Agricultural Sciences, Beijing 100081, P.R. China

§ Both authors contributed equally to this paper.

***Corresponding author**:

Ruifu Zhang , College of Resources & Environmental Sciences, Nanjing Agricultural University, 210095, Nanjing, China; Correspondence should be addressed to R.Z (email: rfzhang@njau.edu.cn).

List of content:

Supplementary Figures: 11

Supplementary Tables: 2

Supplementary Nodes (R Codes): 3

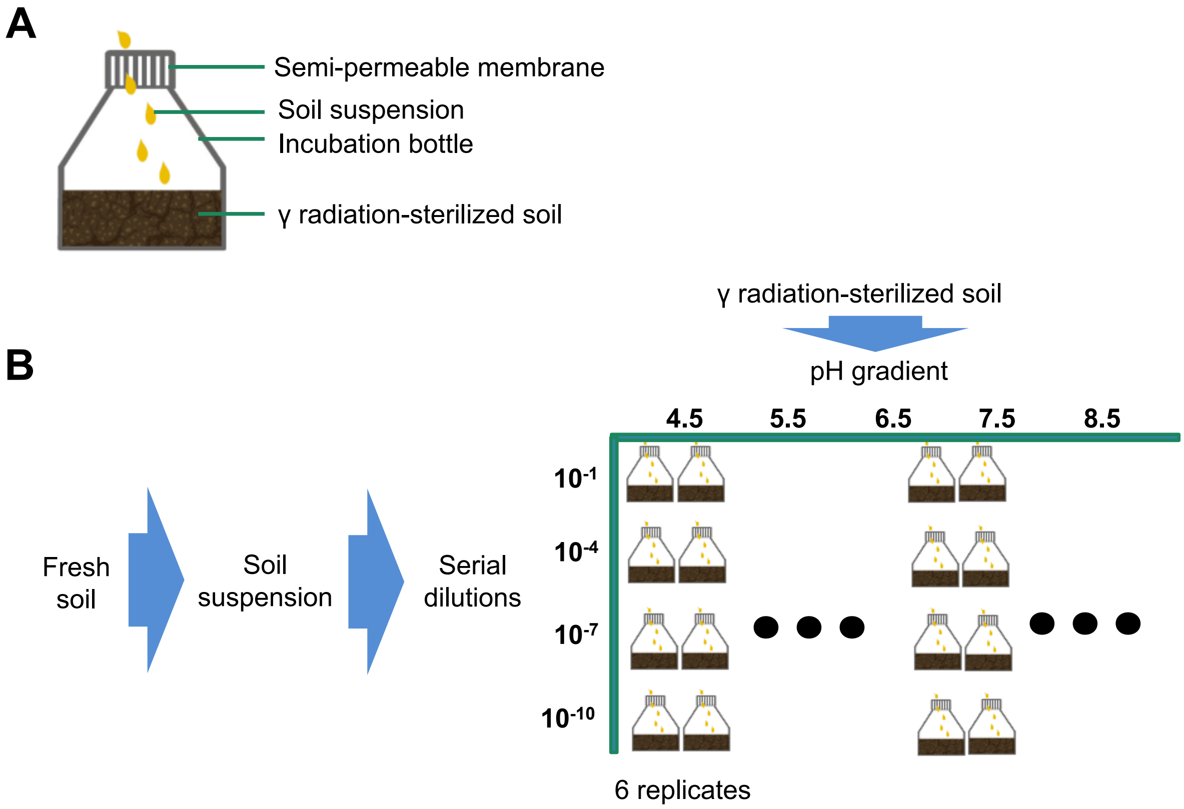

Supplementary Figure S1. (**A**) Schematic diagram of soil microcosm establishment. (**B**) Soil incubation experimental design.

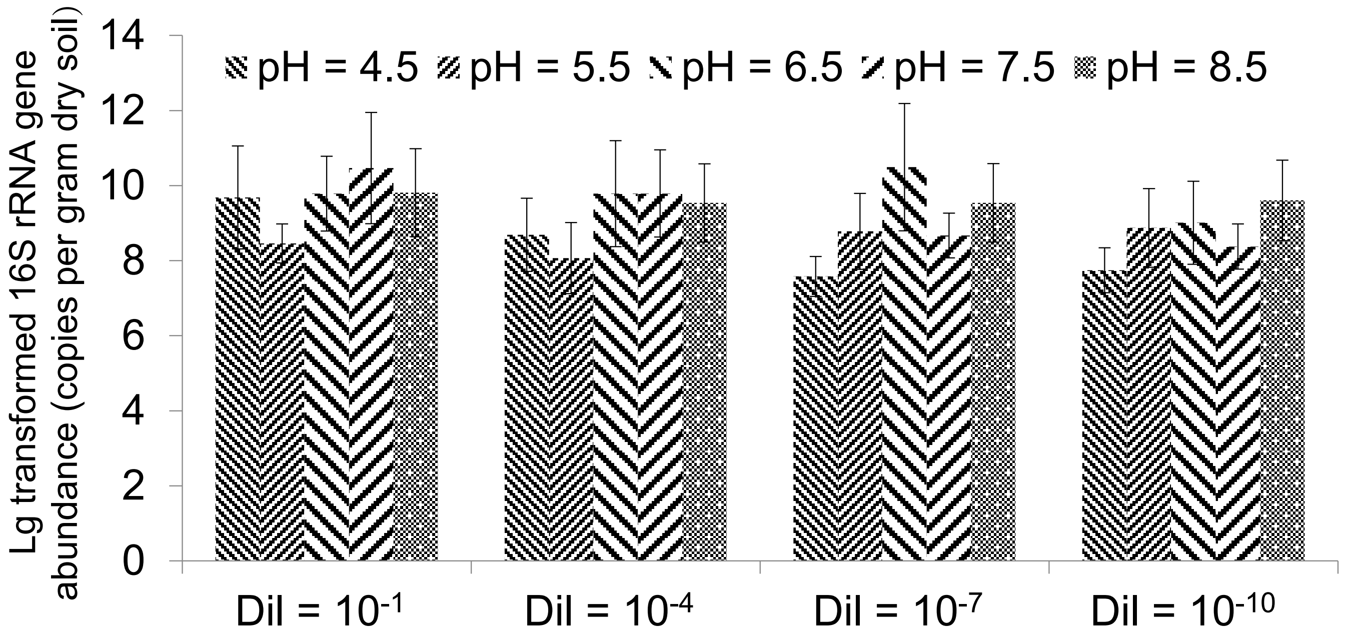

Supplementary Figure S2. The Lg transformed 16S rRNA gene copies in all microcosms at the end of incubation period (sixteen weeks) by quantitative real-time PCR. Error bars represent standard deviations (SD, n = 12). Dil: Dilution level. No statistically significant differences were found among different dilutions and pH levels.

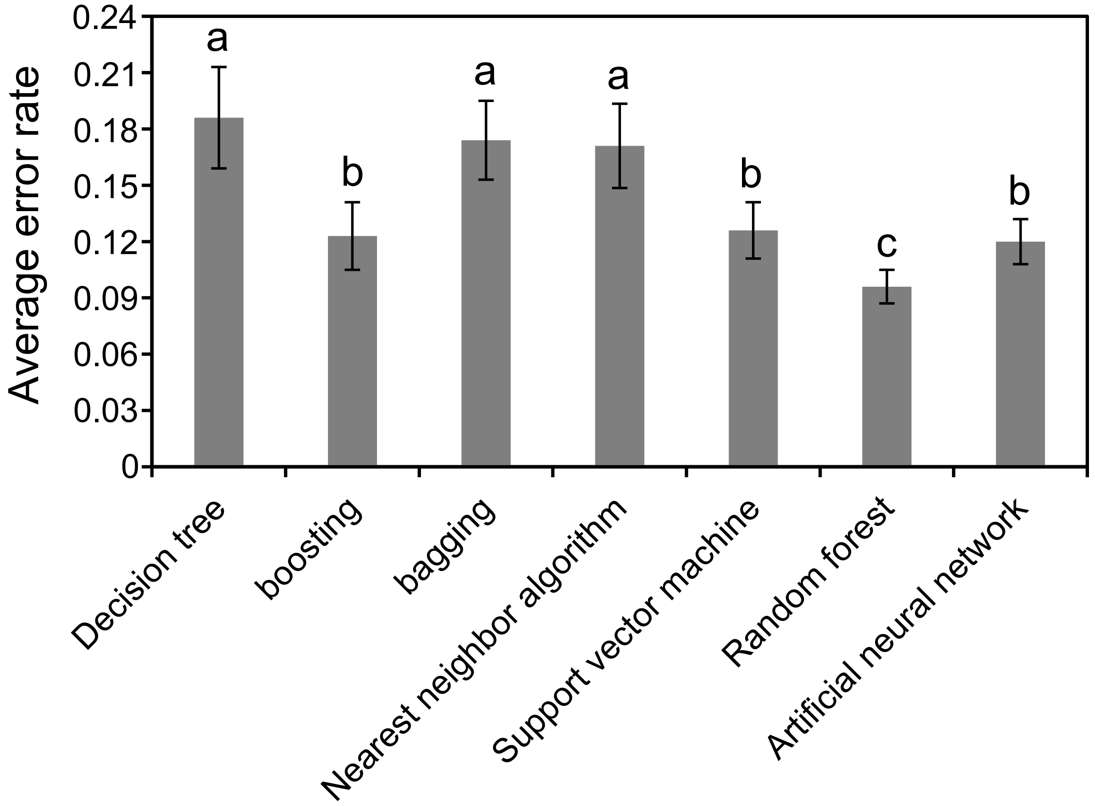

Supplementary Figure S3. The average error rate of 10-fold cross validation based on seven machine learning classification methods. Error bars represent standard deviations (SD, n = 12). Different letters above bars indicate significant differences (*P*-value < 0.05) between machine learning methods according to Duncan's multiple comparison.

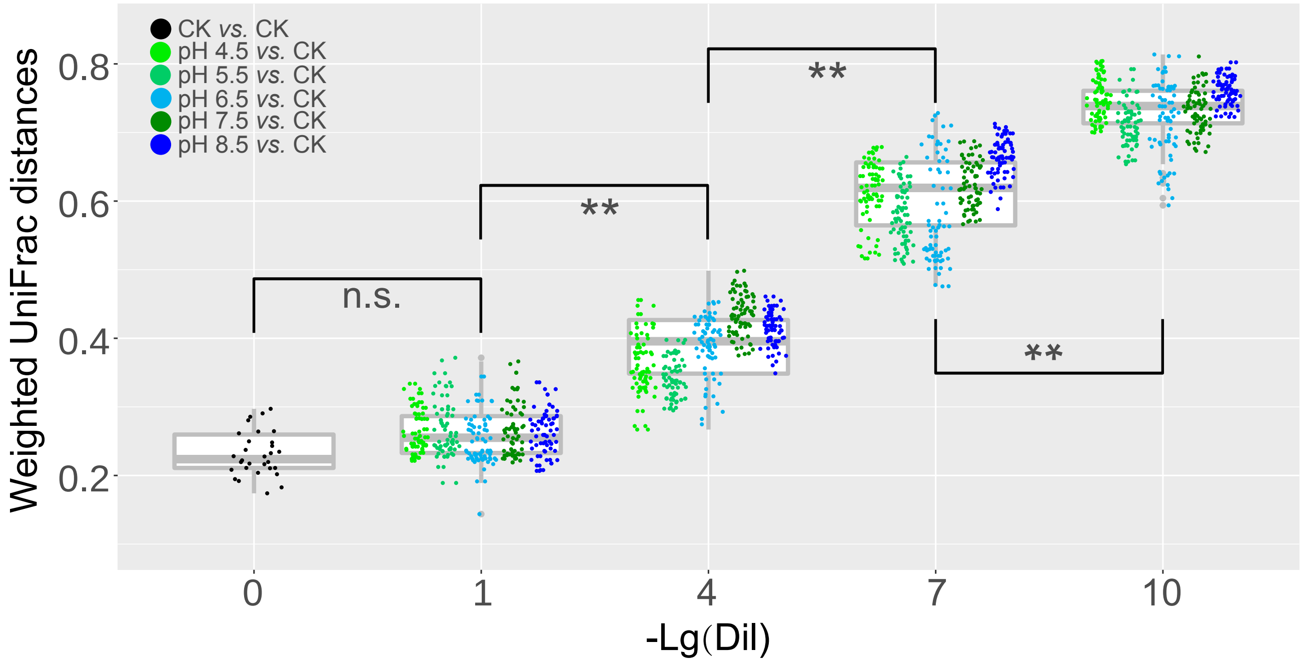

Supplementary Figure S4. Weighted Unifrac distances between bacterial communities from different dilution levels and the untreated initial soil (CK). Lg(Dil) indicates the Lg transformed dilution level. Lg(Dil) = 0 represents the untreated soil. Asterisks indicate significance: **, P-value < 0.01 based on Tukey's HSD test. n.s.: Not significant. Boxplot: median, 25%/75% percentiles, and the highest, lowest and extremely values are shown.

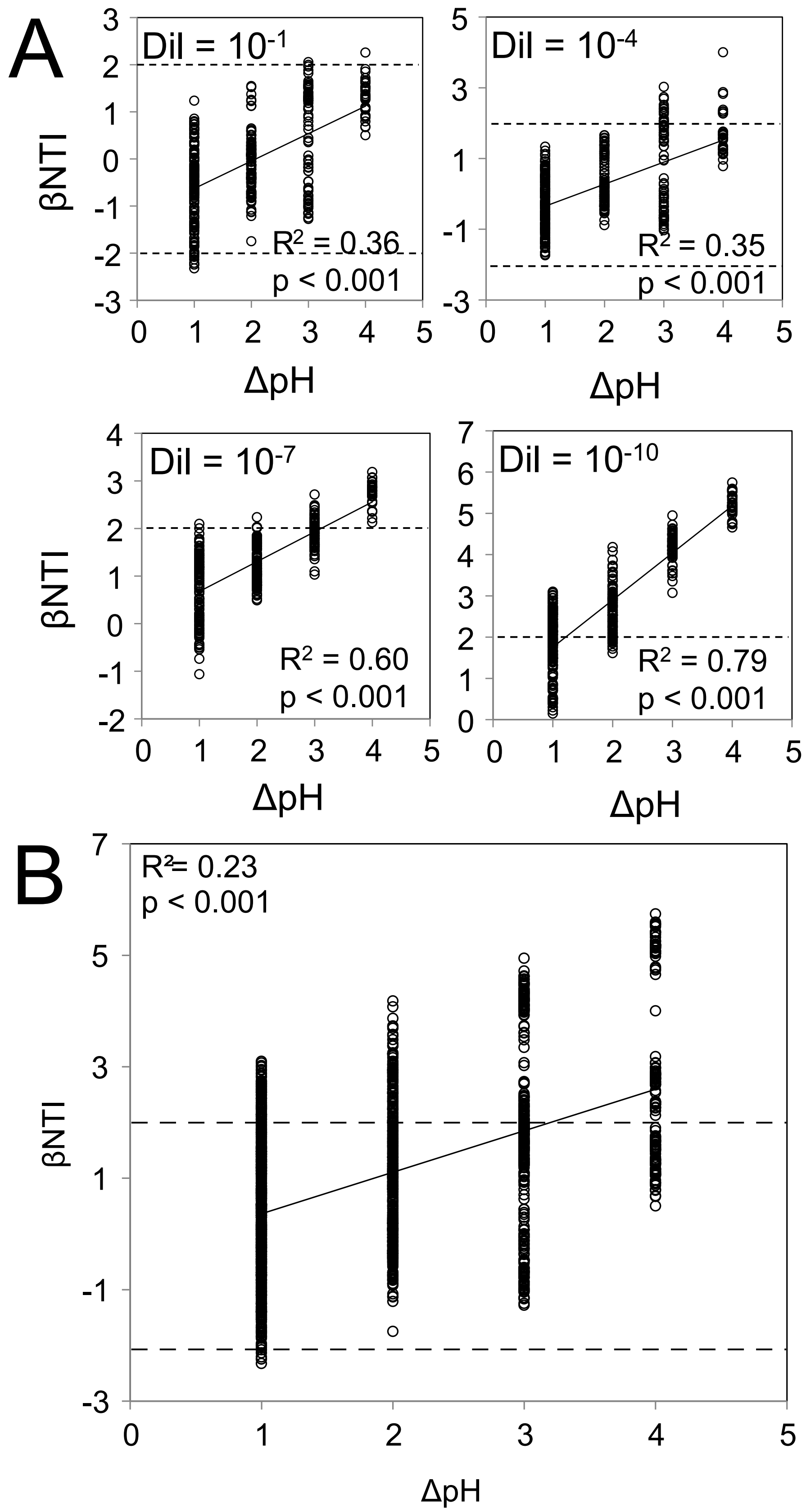

Supplementary Figure S5. The relationship between βNTI and differences in soil pH on each dilution level (**A**) and across all samples (**B**).

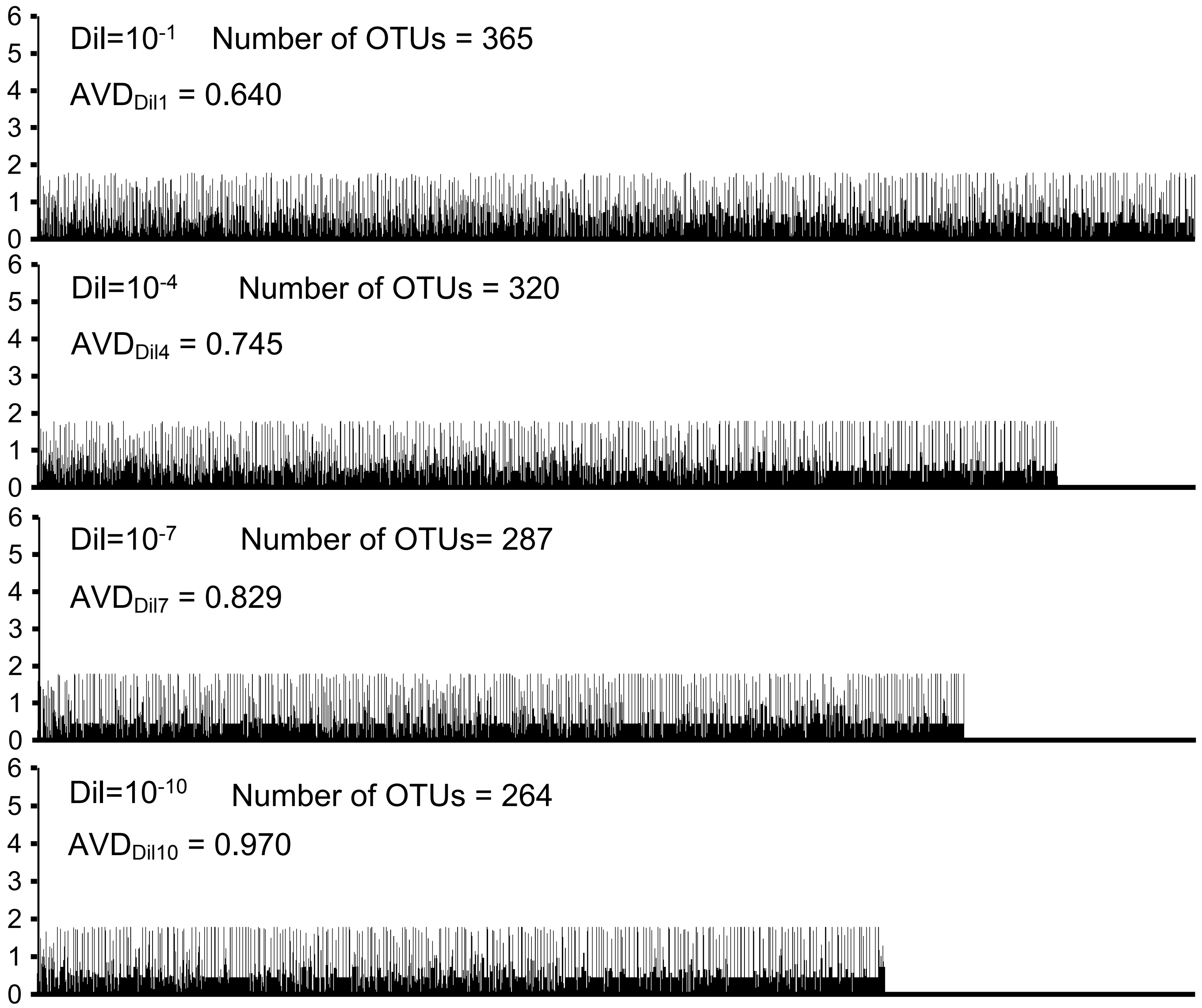

Supplementary Figure S6. The average variation degree (AVD) of all OTUs on each dilution level. Horizontal axis represents different OTUs and vertical axis represents the absolute AVD value of each OTU.

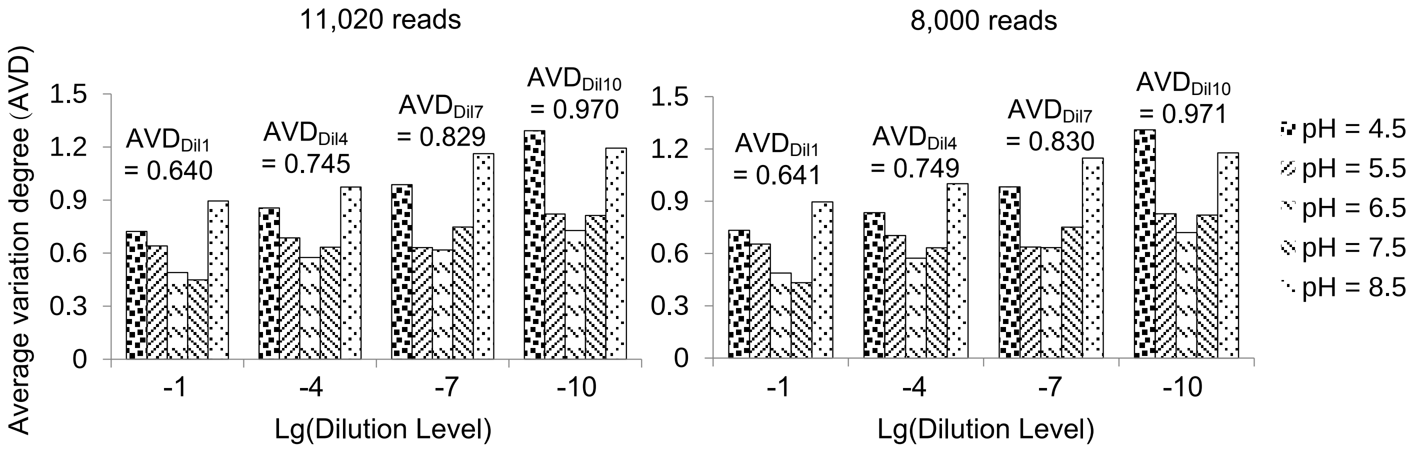

Supplementary Figure S7. The average variation degree (AVD) values under different rarefaction depths of 11,020 reads and 8,000 reads per sample.

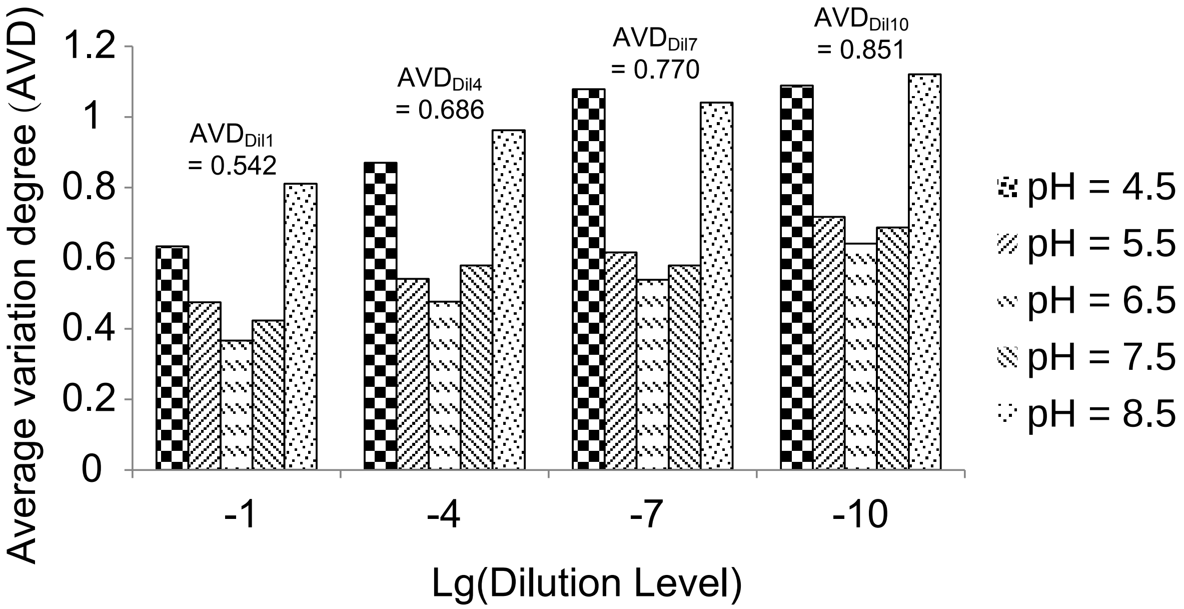

Supplementary Figure S8. The average variation degree (AVD) values under the normalization method of DESeq variance stabilization.

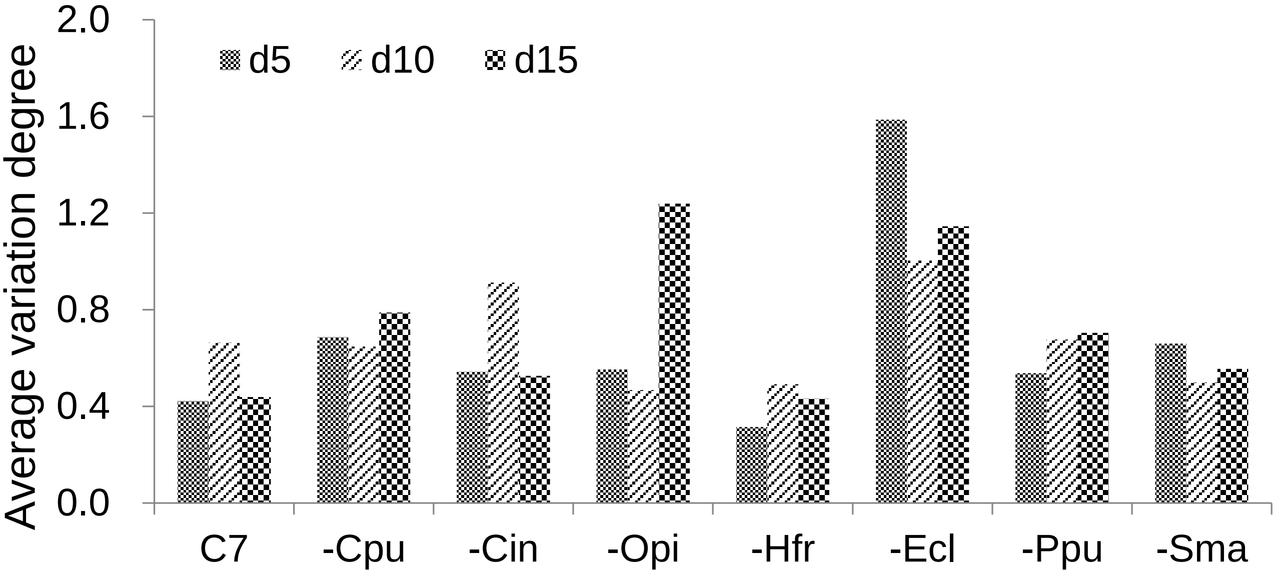

Supplementary Figure S9. The average variation degree (AVD) values of a community stability test (ref 19 of the main text) by detecting the dynamics of community composition with one of the species removed from the original seven-strains synthetic community. The seven strains are *E. cloacae* (Ecl), *S. maltophilia* (Sma), *O. pituitosum* (Opi), *H. frisingense* (Hfr), *P. putida* (Ppu), *C. pusillum* (Cpu), and *C. indologenes* (Cin). C7, all seven strains; -Cpu, remove *C. pusillum* from seven strains; -Cin, remove *C. indologenes* from seven strains; -Opi, remove *O. pituitosum* from seven strains; -Hfr, remove *H. frisingense* from seven strains; -Ecl, remove *E. cloacae* from seven strains; -Ppu, remove *P. putida* from seven strains; -Sma, remove *S. maltophilia* from seven strains. The AVD values of d5, d10 and d15 represent the community compositional variations in the incubation period of 5, 10 and 15 days.

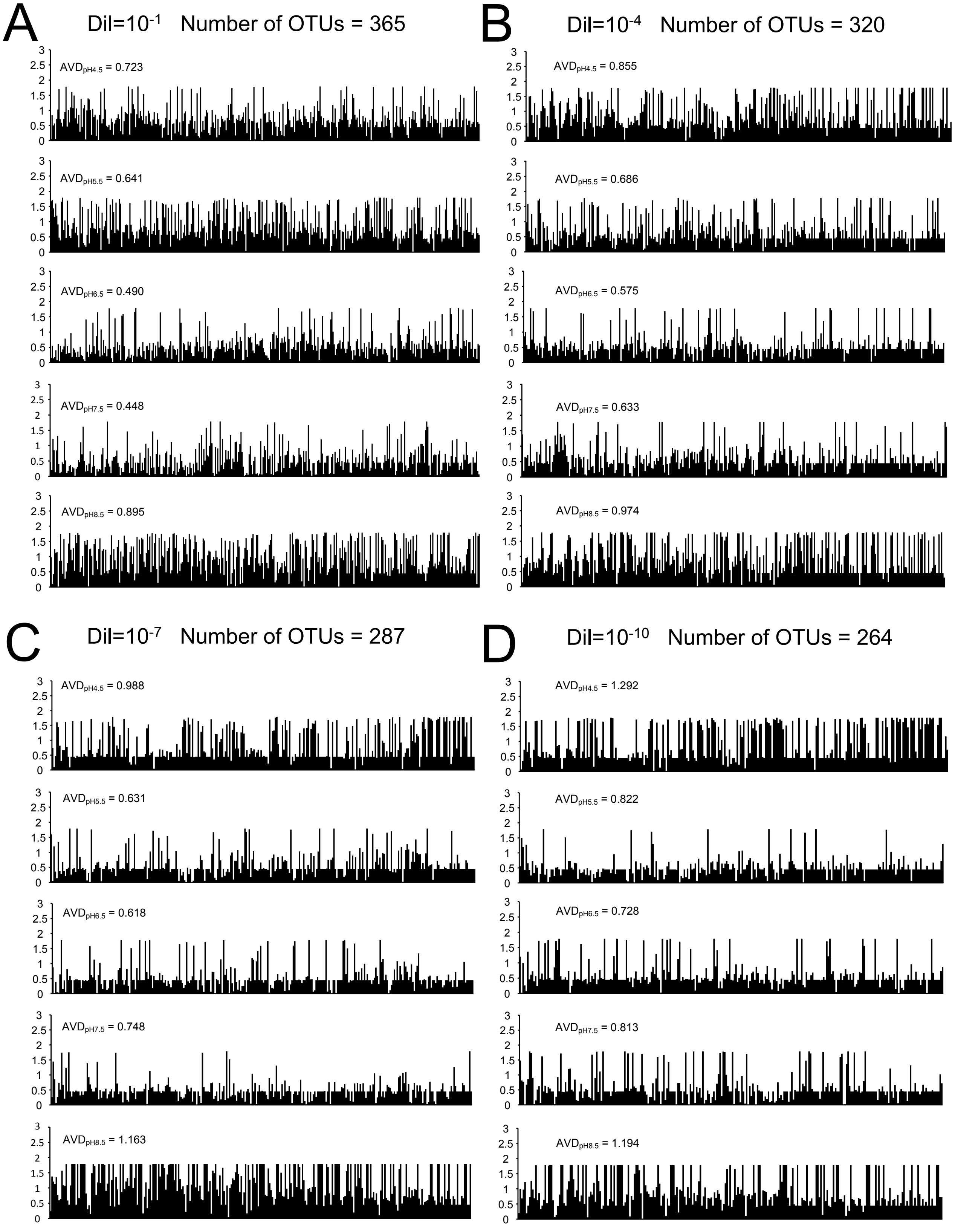

Supplementary Figure S10. The average variation degree (AVD) of all OTUs on each pH level for 10^{-1} (**A**), 10^{-4} (**B**), 10^{-7} (**C**) and 10^{-10} (**D**) diluted samples. Horizontal axis represents different OTUs and vertical axis represents the absolute AVD value of each OTU.

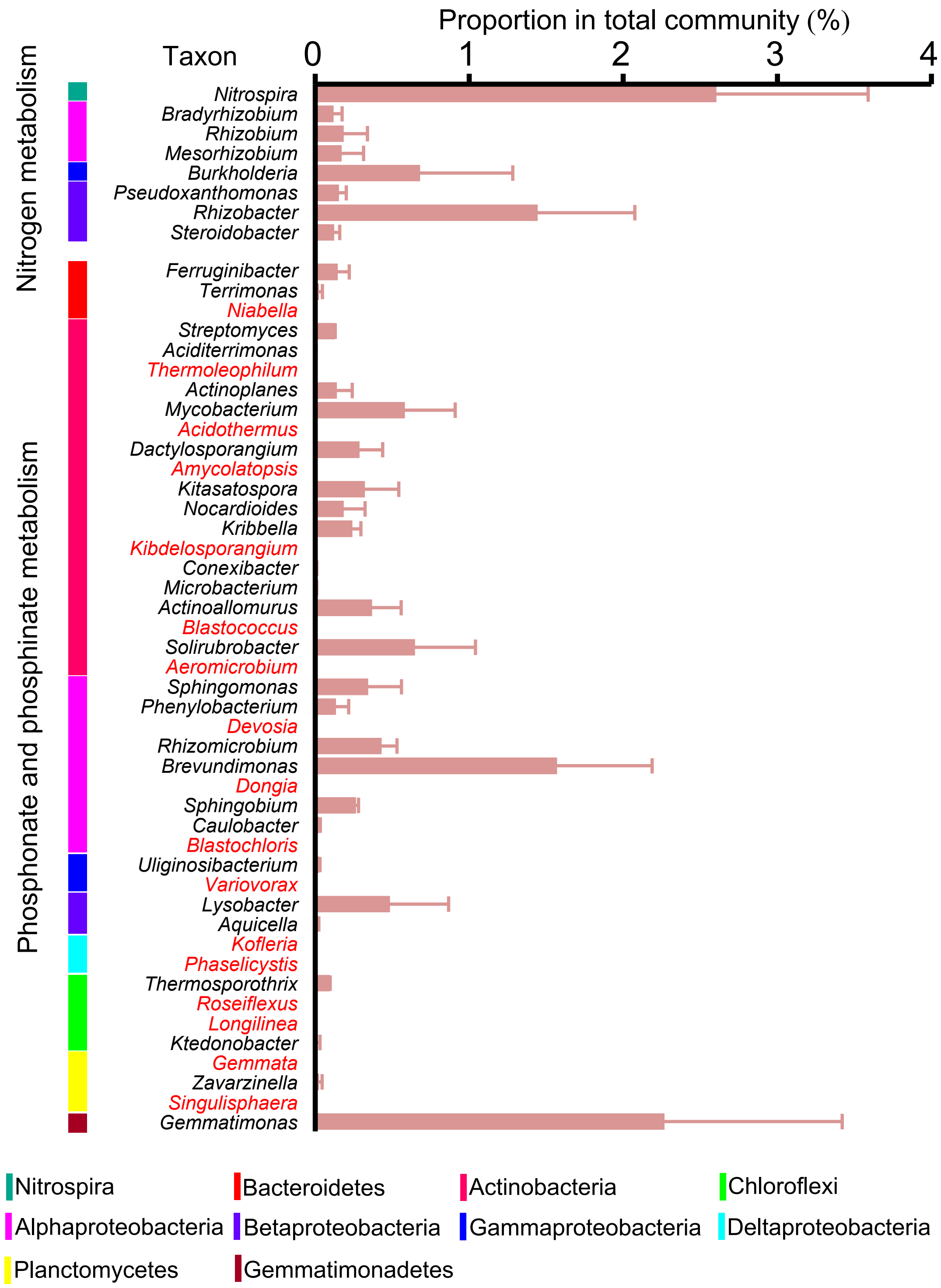

Supplementary Figure S11. The relative abundance of the same bacterial genera in Figure 3 detected by 16S rRNA gene amplicon sequencing. The genera with red text were not detected in amplicon sequencing. Error bars represent standard deviations (SD, n = 240).

Supplementary Table S1. The packages and functions of machine learning classification methods

Classification method	Library	Function	Ref
Decision tree	"rpart"	rpart()	[48]
boosting	"adabag"	boosting()	[49]
bagging	"adabag"	bagging()	[49]
Nearest neighbor algorithm	"kknn"	kknn()	[50]
Support vector machine	"kernlab"	ksvm()	[51]
Random forest	"randomForest"	randomForest()	[52]
Artificial neural network	"nnet"	nnet()	[53]

Supplementary Table S2. The nodes identified as module hubs in the functional gene co-occurrence networks on four dilution levels

Network	Marked node[a]	Functional category 1	Functional category 2	Functional category 3	Topological role	Degree	*Zi*	*Pi*	Clustering coefficient
Dil = 10^{-1}	A	Specialized metabolic function	Energy metabolism	Nitrogen metabolism	Module hub	45	2.73	0.10	0.652
	B	Cellular processes	Cell communication	Adherens junction	Module hub	24	2.63	0.00	0.702
Dil = 10^{-4}	C	Specialized metabolic function	Metabolism of other amino acids	Phosphonate and phosphinate metabolism	Module hub	42	2.75	0.04	0.568
	D	Environmental information processing	Signaling molecules and interaction	Ion channels	Module hub	33	2.83	0.00	0.668
Dil = 10^{-7}	E	Broad metabolic function	Carbohydrate metabolism	Citrate cycle	Module hub	24	2.51	0.02	0.586
	F	Cellular processes	Cell motility	Bacterial chemotaxis	Module hub	12	2.53	0.12	0.503
Dil = 10^{-10}	G	Broad metabolic function	Carbohydrate metabolism	Glycolysis/ Gluconeogenesis	Module hub	23	2.62	0.19	0.675
	H	Broad metabolic function	Carbohydrate metabolism	Starch and sucrose metabolism	Module hub	18	2.61	0.14	0.589

[a] Keystone genes labeled in Figure 2.

Supplementary Note 1 (R codes)

R codes for 10-fold cross validation

```
# function to divide the data into Z parts randomly.
Fold=function(Z=10,Test,Dv,seed=1000){
n=nrow(Test)
d=1:n;dd=list()
e=levels(Test[,Dv])
T=length(e);set.seed(seed) # T is the dependent variable
for(i in 1:T){
d0=d[w[,Dv]==e[i]];j=length(d0)
ZT=rep(1:Z,ceiling(j/Z))[1:j]
id=cbind(sample(ZT,length(ZT)),d0);dd[[i]]=id}
mm=list();for(i in 1:Z){u=NULL;
for(j in 1:T)u=c(u,dd[[j]][dd[[j]][,1]==i,2])
mm[[i]]=u} # mm[[i]] is the ith subscript set: i=1, 2, …, Z
return(mm)} # Output 10 subscript sets
Test=read.csv("Test.csv")
Dv;Z=10;n=nrow(Test);mm=Fold(Z=10,Test,Dv,seed=7777) #Dv is the position of independent variable
```

Classification method:

Decision tree

```
library(rpart)
E=rep(0,Z)
for(i in 1:Z){m=mm[[i]]; # mm[[i]] is the ith subscript set extracted by Fold() function: i=1, 2, …, Z
n1=length(m);a=rpart(avd~.,Test[-m,]) # avd is the independent variable
E[i]=sum(Test[m,Dv]!=predict(a,Test[m,])$avd)/n1}
mean(E)
```

boosting

```
library(adabag)
E=rep(0,Z)
for(i in 1:Z){m=mm[[i]]; # mm[[i]] is the ith subscript set extracted by Fold() function: i=1, 2, …, Z
n1=length(m)
a=boosting(avd~.,Test[-m,]) # avd is the independent variable
E[i]=sum(as.character(Test[m,Dv])!=predict(a,Test[m,])$avd)/n1}
mean(E)
```

bagging

```
library(adabag)
E=rep(0,Z)
for(i in 1:Z){m=mm[[i]]; # mm[[i]] is the ith subscript set extracted by Fold() function: i=1, 2, …, Z
n1=length(m)
a=bagging(avd~.,Test[-m,]) # avd is the independent variable
E[i]=sum(as.character(Test[m,Dv])!=predict(a,Test[m,])$avd)/n1}
mean(E)
```

Nearest neighbor algorithm

```
library(kknn)
E=rep(0,Z)
for(i in 1:Z){m=mm[[i]]; # mm[[i]] is the ith subscript set extracted by Fold() function: i=1, 2, …, Z
n1=length(m)
a=kknn(avd~.,k=6,train=Test[-m,],test=Test[m,]) # avd is the independent variable
E[i]=sum(Test[m,Dv])!=a$avd/n1}
mean(E)
```

Support vector machine

```
library(kernlab)
E=rep(0,Z)
for(i in 1:Z){m=mm[[i]]; # mm[[i]] is the ith subscript set extracted by Fold() function: i=1, 2, …, Z
n1=length(m)
a=ksvm(avd~.,data=Test[-m,]) # avd is the independent variable
E[i]=sum(Test[m,Dv]!=predict(a,Test[m,]))/n1}
mean(E)
```

Random forest

```
library(randomForest)
E=rep(0,Z)
for(i in 1:Z){m=mm[[i]]; # mm[[i]] is the ith subscript set extracted by Fold() function: i=1, 2, …, Z
n1=length(m)
a=randomForest(avd~.,data=Test[-m,]) # avd is the independent variable
E[i]=sum(Test[m,Dv]!=predict(a,Test[m,]))/n1}
mean(E)
```

Artificial neural network

```
library(nnet)
E=rep(0,Z)
for(i in 1:Z){m=mm[[i]]; # mm[[i]] is the ith subscript set extracted by Fold() function: i=1, 2, …, Z
n1=length(m)
mc=setdiff(1:n,m)
a=nnet(avd~.,data=Test,subset=mv,size=7,range=0.1,decay=0.01,maxit=200) # avd is the independent variable
E(i)=sum(Test[m,Dv]!=predict(a,Test[m,])$avd)/n1}
mean(E)
```

Functional importance:

(An example dataset has been provided in supplementary data files)

```
library(randomForest)
library(ggplot2)
library("caret")
# divide the data set into training set and test set
ind = sample(2,nrow(Test),replace = TRUE, prob = c(0.7,0.3))
set.seed(1000)
Test.train = Test[ind == 1,]
Test.test = Test[ind == 2,]
#find the optimal mtry
```

```
n<-length(names(Test.train))
min=100
num=0
for (i in 1:(n-1)){
mtry_fit<-randomForest(avd~., data=Test.train, mtry=i)
err<-mean(mtry_fit$err.rate)
print(err)
if(err<min) {
min =err
num=i }
  }
print(min)
print(num) # num is the optimal mtry
#find the optimal ntree
ntree_fit<-randomForest(avd~.,data=Test.train,mtry=num,ntree=1000)
plot(ntree_fit)
# feature importance
a<-randomForest(avd~.,Test, mtry=num,ntree=ntree_optimal,importance=TRUE) # avd is the independent variable, mtry is the selected optimal mtry value, ntree_optimal is the selected optimal ntree value.
barplot(importance(a),cex.name=0.6)
title("Functional importance")
```

Supplementary Note 2 (R codes)

R codes for calculating spearman’s correlation between gene categories

```
data <- read.table("gene_abundance.txt",header = TRUE,row.names = 1,sep = "\t")
# read in a gene abundance  table
data <- t(data)
library(psych)
occor =corr.test(data,method = "spearman",adjust = " fdr") # calculate spearman’s
correlation
occor.r = occor$r # extract r-value of spearman’s correlation
occor.p = occor$p # extract p-value of spearman’s correlation
occor.r[occor.p>0.01|abs(occor.r)<0.75] = 0 # r-value and p-value filtering
write.table(occor.r,"pvalue.csv",sep="\t",quote=F,col.names=NA) # output results
```

Supplementary Note 3 (R codes)

R codes for calculating βNTI metrics

(An example dataset has been provided in supplementary data files)

```
# Reconstructing Phylogenetic Trees （Linux environment）
mafft --maxiterate 1000 --auto otutab.fa >otutab_aligned.fa
fasttree -gtr -nt otutab_aligned.fa >otutab.nwk
# Calculate BetaNTI (R environment)
getwd()
library(picante)
data.set.name = 'otutab'
otu = as.data.frame(read.table(paste(data.set.name,".txt",sep=""),header=T,row.names=1));
dim(otu);
otu=t(otu);
dim(otu)
phylowb = read.tree(paste(data.set.name,".nwk",sep=""))
phylowb
match.phylowb.otu = match.phylo.data(phylowb, t(otu))
str(match.phylowb.otu)
write.tree(match.phylowb.otu$phy,paste(data.set.name,".tre",sep=""))
phylowbMatch = read.tree(paste(data.set.name,".tre",sep=""));
phylowbMatch
match.phylowbMatch.otu = match.phylo.data(phylowbMatch, t(otu))
str(match.phylowbMatch.otu)
beta.mntd.weighted = as.matrix(comdistnt(t(match.phylowbMatch.otu$data),
cophenetic(match.phylowbMatch.otu$phy), abundance.weighted=T))
beta.mntd.weighted[1:5,1:5]
write.csv(beta.mntd.weighted,'betaMNTD_weighted.csv',quote=F)
identical(colnames(match.phylowbMatch.otu$data),colnames(beta.mntd.weighted)); # Just checking
and should be TRUE
identical(colnames(match.phylowbMatch.otu$data),rownames(beta.mntd.weighted)); # Just checking
and should be TRUE
beta.reps = 999; # number of randomizations
random.weighted.bMNTD.comp =
array(c(-999),dim=c(ncol(match.phylowbMatch.otu$data),ncol(match.phylowbMatch.otu$data),beta.re
ps))
dim(random.weighted.bMNTD.comp);

for (rep in 1:beta.reps) {

random.weighted.bMNTD.comp[,,rep] =
as.matrix(comdistnt(t(match.phylowbMatch.otu$data),taxaShuffle(cophenetic(match.phylowbMatch.ot
u$phy)),abundance.weighted=T,exclude.conspecifics = F))
```

```
print(c(date(),rep));
}

weighted.bNTI =
matrix(c(NA),nrow=ncol(match.phylowbMatch.otu$data),ncol=ncol(match.phylowbMatch.otu$data))

dim(weighted.bNTI)

for (columns in 1:(ncol(match.phylowbMatch.otu$data)-1)) {
    for (rows in (columns+1):ncol(match.phylowbMatch.otu$data)) {

        random.vals = random.weighted.bMNTD.comp[rows,columns,];
        weighted.bNTI[rows,columns] = (beta.mntd.weighted[rows,columns] - mean(random.vals)) /
sd(random.vals);
        rm("random.vals");

    };
};

rownames(weighted.bNTI) = colnames(match.phylowbMatch.otu$data);
colnames(weighted.bNTI) = colnames(match.phylowbMatch.otu$data);
weighted.bNTI;
write.csv(weighted.bNTI,"weighted_bNTI.csv",quote=F);
```
